# The Relationship Between Blood Urea Nitrogen to Albumin Ratio (BAR) and Diabetic Foot Ulcer (DFU) in Diabetic Patients

**DOI:** 10.3390/healthcare14142209

**Published:** 2026-07-21

**Authors:** Zeyang Yu, Tianbo Li, Jiangning Wang, Lei Gao

**Affiliations:** Department of Orthopedics Surgery, Capital Medical University Affiliated Beijing Shijitan Hospital, No. 10 Yangfangdian Tieyi Road, Haidian District, Beijing 100038, China; yuzeyang7432@bjsjth.cn (Z.Y.); litianbo3285@bjsjth.cn (T.L.); wangjn@bjsjth.cn (J.W.)

**Keywords:** blood urea nitrogen to albumin ratio, diabetic foot ulcer, NHANES, risk factor, cross-sectional study

## Abstract

**Objective:** To examine the association between the blood urea nitrogen-to-albumin ratio (BAR) and diabetic foot ulcer (DFU) among individuals with diabetes, and to determine whether BAR may provide additional value for DFU risk assessment. **Methods:** Data were obtained from diabetic participants in the National Health and Nutrition Examination Survey (NHANES) 1999–2004. A total of 1337 eligible individuals were included in the final analysis. BAR was calculated by dividing serum urea nitrogen concentration (mg/dL) by serum albumin concentration (g/dL). DFU status was identified according to self-reported questionnaire information. Logistic regression models with progressive adjustment were used to evaluate the association between BAR and DFU. Restricted cubic spline analysis was performed to explore the dose–response pattern, and stratified analyses were conducted to assess whether the association varied across different clinical and demographic subgroups. **Results:** After full adjustment for potential confounders, higher BAR was significantly associated with increased odds of DFU. Each one-unit increase in BAR corresponded to a 21% higher risk of DFU (OR = 1.21, 95% CI: 1.09–1.35, *p* < 0.001). In the quartile-based analysis, participants in the highest BAR quartile had a markedly greater risk of DFU than those in the lowest quartile (OR = 3.11, 95% CI: 1.09–8.92, *p* = 0.035). Restricted cubic spline analysis indicated a non-linear relationship between BAR and DFU risk (*p* = 0.0127). Subgroup analyses showed that the positive association was generally consistent across strata defined by sex, body mass index, smoking status, and hypertension, with no significant interaction observed (all *p* for interaction > 0.05). **Conclusions:** Elevated BAR was independently associated with a higher risk of DFU among diabetic individuals. As an easily accessible composite index reflecting renal function, nutritional status, and systemic inflammatory burden, BAR may help identify diabetic patients who are more likely to develop foot ulceration and may support earlier preventive management.

## 1. Introduction

Diabetic foot ulcer (DFU) represents one of the most severe and devastating complications of diabetes, and has emerged as the leading cause of non-traumatic lower extremity amputations [1,2]. It not only significantly impairs patients’ quality of life but also imposes a substantial socio-medical burden. The progression of DFU creates a highly complex microenvironment characterized by chronic infection, hyperglycemia, hypoxia, impaired blood flow, and severe inflammation [3,4]. It is reported that DFU affects approximately 6.3% of diabetic patients. Approximately 20% of diabetic foot ulcers (DFUs) complicated by moderate-to-severe infection progress to lower-extremity amputation, with a post-amputation five-year mortality rate exceeding 70% [1,5,6]. In the United States alone, approximately 150,000 patients are hospitalized annually due to DFU, incurring healthcare costs exceeding $31 billion, underscoring the severity of this condition as a public health issue [7]. The pathogenesis of DFU is multifactorial, involving chronic hyperglycemia-induced multisystem dysfunction, including peripheral neuropathy, vasculopathy, persistent inflammation, and immune dysregulation. These factors collectively contribute to impaired tissue repair capacity and poor ulcer healing [1,4].

Strict glycemic control, including the monitoring of glycated hemoglobin (HbA1c), remains a cornerstone of diabetes management; in particular, elevated HbA1c levels have been identified as a significant factor associated with the occurrence of diabetic foot [8]. However, glycemic indicators alone have notable limitations in predicting DFU risk because they do not fully reflect nutritional status, systemic inflammation, renal function, or microvascular injury. While HbA1c reflects the average blood glucose level over the preceding 2 to 3 months [9], it does not comprehensively capture key influencing factors such as nutritional status, local or systemic inflammation, and renal function. However, inflammation and renal function play critical roles in the development and progression of DFU [10,11,12]. Consequently, there is an urgent clinical need for novel biomarkers that integrate multiple pathological pathways to enable more accurate risk stratification and early intervention.

In recent years, the Blood Urea Nitrogen to Albumin Ratio (BAR) has garnered attention as a composite biomarker. BAR concurrently reflects renal function via serum urea nitrogen and nutritional/inflammatory status via serum albumin [13]. Elevated urea nitrogen often indicates impaired renal function or increased protein catabolism, while low albumin levels are closely associated with chronic inflammation, exacerbated oxidative stress, and diminished tissue repair capacity [14]. BAR is calculated as the ratio of serum urea nitrogen (mg/dL) to serum albumin (g/dL). This metric integrates both metabolic and inflammatory information, potentially offering superior predictive value compared to individual parameters. Previous studies have demonstrated the prognostic utility of BAR in cardiovascular diseases, acute kidney injury, and other conditions [15]. However, its role in DFU risk assessment remains underexplored in large-scale population studies. In particular, current biomarker research in DFU has predominantly focused on single indicators [16], with limited epidemiological evidence on composite parameters such as BAR. Its specific association in diabetic populations has not been clearly established.

Albumin and blood urea nitrogen, the two components of BAR, are biologically relevant to DFU. Hypoalbuminemia may reflect malnutrition and systemic inflammation, both of which can impair wound repair, whereas elevated blood urea nitrogen may indicate renal dysfunction and microvascular injury. Therefore, BAR may capture the combined influence of nutritional status, inflammation, and renal dysfunction on DFU risk [12,13,14].

Therefore, this study, based on nationally representative data from the National Health and Nutrition Examination Survey (NHANES) 1999–2004, proposes the following core hypothesis: elevated BAR is positively associated with the prevalence of DFU, independent of traditional risk factors such as age, blood glucose level, and hypertension. The primary objectives include: (1) analyzing the strength of association between BAR—as both a continuous variable and a quartile-based categorical variable—and DFU; (2) exploring potential non-linear relationships between BAR and DFU using restricted cubic spline (RCS) models; (3) assessing the consistency of this association across various subgroups (e.g., by sex, body mass index, smoking status); and (4) providing new biomarker-based evidence for early identification and risk stratification of DFU. Through this cross-sectional study, we aim to address the knowledge gap regarding BAR in diabetic complication research and provide a theoretical foundation for future interventional studies targeting nutritional support or anti-inflammatory therapies.

## 2. Method

### 2.1. Study Population

**Figure 1:**
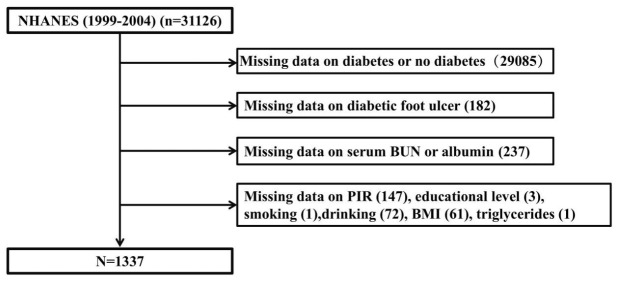
The flow diagram of the study participants. PIR, poverty-to-income ratio; BMI, body mass index. BUN, blood urea nitrogen.

Data for this analysis were drawn from the 1999–2004 cycles of the National Health and Nutrition Examination Survey (NHANES). NHANES is an ongoing population-based survey administered by the National Center for Health Statistics (NCHS), designed to collect health, nutritional, laboratory, and questionnaire information from the civilian non-institutionalized U.S. population. The datasets and accompanying documentation are publicly available at https://www.cdc.gov/nchs/nhanes/ (accessed on 10 September 2025). In the present study, participants were excluded if they had no available diabetes-related information, did not meet the definition of diabetes, lacked questionnaire data for DFU status, had missing measurements of serum urea nitrogen or serum albumin, or had incomplete information on covariates required for the adjusted analyses. The detailed process of sample selection is shown in Figure 1.

### 2.2. Definition of Bar, Diabetes and Diabetic Foot Ulcers

BAR was calculated based on laboratory measurements from NHANES. The formula used was: BAR = serum urea nitrogen (mg/dL) divided by serum albumin (g/dL). Participants were classified as having diabetes if they met any of the following criteria: (1) glycated hemoglobin (HbA1c) level ≥ 6.5%; (2) fasting plasma glucose ≥ 7.0 mmol/L; (3) random blood glucose ≥ 11.1 mmol/L; (4) self-reported physician-diagnosed diabetes; or (5) current use of oral hypoglycemic agents or insulin. DFU was assessed using a questionnaire. Participants were asked: “Have you ever had an ulcer or sore on your leg or foot that took more than 4 weeks to heal?” Those who answered “yes” were defined as having DFU, while those who answered “no” were defined as not having DFU. DFU status was assessed using a self-reported questionnaire item from NHANES, defined as a history of foot or leg ulcer that took more than 4 weeks to heal, rather than clinically confirmed active diabetic foot ulcer at the time of examination.

### 2.3. Covariates

The covariates considered in this study included age, sex, race/ethnicity, educational attainment, poverty-to-income ratio (PIR), body mass index (BMI), smoking status, alcohol intake, hypertension, triglycerides, total cholesterol, and HbA1c. Race/ethnicity was grouped as Mexican American, other Hispanic, non-Hispanic White, non-Hispanic Black, and other race. Educational level was classified into two categories: below high school and high school or above. BMI was divided into non-obese and obese groups using 30 kg/m^2^ as the cutoff. Smoking status was categorized as never, former, or current smoking, while alcohol consumption was recorded as yes or no. BMI was measured during the mobile examination center (MEC) visit by trained staff and calculated as weight in kilograms divided by height in meters squared. Participants who reported consuming more than 12 alcoholic drinks per year were defined as alcohol drinkers. Hypertension was considered present if participants reported a physician diagnosis, had a measured blood pressure of at least 140/90 mmHg, or were currently using antihypertensive drug.

### 2.4. Statistic Analysis

Sampling weights from the MEC examination were applied in all statistical analyses to ensure national representativeness. Sampling weights from the MEC examination were applied in all analyses to account for the complex multistage sampling design of NHANES. For categorical variables, unweighted counts and survey-weighted percentages were reported. Baseline characteristics of the study participants were stratified by the presence or absence of DFU. Continuous variables are presented as mean ± standard deviation, whereas categorical variables are expressed as numbers and percentages (%). Differences between groups were evaluated using Pearson’s chi-square test for categorical variables and the Kruskal–Wallis test for continuous variables. For categorical variables, differences between groups were evaluated using Pearson’s chi-square test with the Rao & Scott adjustment to account for the complex multistage sampling design of NHANES. Logistic regression models were employed to examine the association between BAR—analyzed both as a continuous variable and in quartiles—and the risk of DFU. Three models were constructed: Model 1 was unadjusted; Model 2 was adjusted for age, gender, race, PIR, education, BMI, smoking, and drinking; and Model 3 included additional adjustments for hypertension, triglycerides, total cholesterol, and HbA1c. To examine potential non-linear relationships between BAR and DFU risk, a restricted cubic spline (RCS) model was applied with multivariable adjustment as in Model 3. Subgroup analyses were conducted to evaluate the consistency of the association across strata defined by gender, educational level, BMI, smoking status, alcohol consumption, and hypertension.

All statistical analyses were performed using R software (version 4.4.3). All statistical tests were two-sided, and a *p*-value of <0.05 was considered statistically significant.

## 3. Result

### 3.1. Baseline Characteristics of the Study Participants

As shown in Table 1, a total of 1337 diabetic participants were included based on the inclusion and exclusion criteria, among whom 100 (8.2%) had DFU. Comparison between the DFU and non-DFU groups revealed no significant differences in demographic characteristics such as age, gender, educational attainment, or PIR. However, racial distribution differed significantly between the two groups, with a lower proportion of Mexican Americans and a higher proportion of Non-Hispanic Whites in the DFU group. The prevalence of obesity was significantly higher in the DFU group. No statistically significant differences were observed between the two groups in smoking status, alcohol consumption, prevalence of hypertension, triglycerides, total cholesterol, or glycemic control (*p* > 0.05).

### 3.2. The Relationship Between Bar and Dfu Risks

Results from Table 2 demonstrate a significant positive association between BAR and the risk of DFU. In the crude model (Model 1), each unit increase in BAR was associated with a 17% increase in the risk of DFU (OR = 1.17, 95% CI: 1.09–1.26, *p* < 0.001). This association was strengthened in Model 2 (OR = 1.21, 95% CI: 1.08–1.34, *p* = 0.001) and remained stable in the fully adjusted Model 3 (OR = 1.21, 95% CI: 1.09–1.35, *p* < 0.001). When analyzed by quartiles of BAR, compared with the lowest quartile (Q1, reference), the highest quartile (Q4) showed significantly increased risk of DFU across all three models (*p* < 0.05). It is noteworthy that the second quartile (Q2) showed a significantly reduced risk in Model 1 (OR = 0.29, 95% CI: 0.09–0.93, *p* = 0.039), but this protective effect was no longer significant after further adjustment for covariates. The third quartile (Q3) did not show a significant association in any model.

### 3.3. Rcs Curve

**Figure 2:**
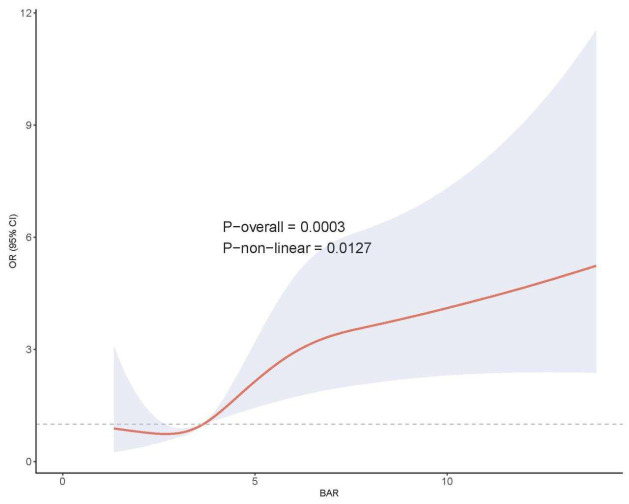
The RCS curve showed a dose response between BAR and the risk of DFU.

To examine a potential non-linear relationship between BAR and DFU, a restricted cubic spline model was fitted. The results indicated a non-linear association between BAR and DFU risk (*p* for non-linearity = 0.0127). The curve demonstrated a gradual increase in DFU risk with rising BAR levels, which is consistent with the findings from the logistic regression analyses (Figure 2).

### 3.4. Subgroup Analysis

**Figure 3:**
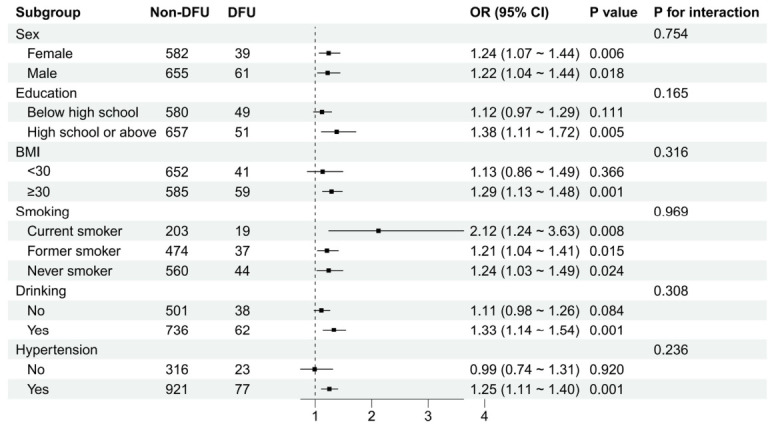
Subgroup analysis of the association between BAR and the risk of DFU.

Subgroup analyses were performed to examine whether the association between BAR and DFU differed across selected participant characteristics. The positive relationship between BAR and DFU was observed across strata defined by sex, educational level, BMI, smoking status, alcohol intake, and hypertension. No significant interaction was detected for these variables, with all interaction *p* values greater than 0.05. These findings indicate that the association between higher BAR and increased DFU risk was broadly consistent across the examined subgroups, although further studies are still needed to confirm its generalizability. The results are shown in Figure 3.

## 4. Discussion

The present analysis focused on adults with diabetes from the 1999–2004 NHANES cycles and explored whether the blood urea nitrogen-to-albumin ratio (BAR) was associated with DFU. Few studies have examined BAR in relation to DFU risk in a large-scale general population. BAR was positively associated with DFU when analyzed as a continuous variable and when categorized into quartiles. The association remained significant after adjustment for demographic characteristics, lifestyle factors, comorbidities, and laboratory parameters. Restricted cubic spline analysis indicated a non-linear relationship between BAR and DFU risk. Subgroup analyses showed comparable associations between BAR and DFU across groups stratified by sex, education level, BMI, smoking status, alcohol intake, and hypertension, with no significant interactions observed. These findings suggest that BAR may serve as a supplementary marker for DFU risk assessment in diabetic patients.

As a composite indicator, BAR has the advantage of simultaneously reflecting renal function through blood urea nitrogen and systemic inflammation or nutritional status through albumin. The results of this study suggest that an elevated BAR may represent a state of metabolic–inflammatory-renal dysfunction, which is a key pathological basis for the development and progression of DFU [17,18]. Elevated urea nitrogen levels are often associated with impaired renal function, enhanced protein catabolism, or dehydration, while low albumin levels typically indicate chronic inflammation, increased oxidative stress, or malnutrition. The combined effect of these factors may lead to reduced tissue repair capacity, immune dysfunction, and microcirculatory disturbances, thereby promoting the occurrence of DFU [13,14]. Previous studies have demonstrated its prognostic value in several clinical conditions, although evidence regarding its association with DFU remains limited. Therefore, the present study provides novel evidence supporting a positive association between BAR and DFU.

It is noteworthy that a significant association between BAR and DFU was still observed in the fully adjusted model (OR = 1.21, 95% CI: 1.08–1.34, *p* = 0.001). This indicates that the predictive ability of BAR for DFU risk is independent of traditional risk factors such as age, glycemic control as measured by HbA1c, hypertension, and lipid levels. This suggests that BAR may capture pathophysiological information not covered by these conventional indicators, particularly regarding inflammatory and nutritional status [10,11,12]. Therefore, BAR has the potential to serve as a supplementary biomarker for a more comprehensive assessment of DFU risk.

Consistent with the findings of this study, the predictive value of BAR in other diseases has gradually been revealed. In a study on diabetic retinopathy, Dai et al. found that BAR was nearly linearly positively associated with the risk of diabetic retinopathy (OR = 1.46, 95% CI: 1.20–1.79, *p* = 0.0002), and this association remained significant after multiple adjustments [19]. This indicates that BAR may have a universal predictive value for various diabetic microvascular complications. Moreover, BAR has also demonstrated good prognostic ability in cardiovascular diseases, acute kidney injury, and other conditions [15,20], further supporting its potential as a cross-disease risk marker [21].

From a mechanistic perspective, an elevated BAR may promote the occurrence of DFU through multiple pathways. On one hand, high urea nitrogen may reflect a decline in glomerular filtration rate, leading to the accumulation of uremic toxins, which further inhibits fibroblast proliferation, collagen synthesis, and angiogenesis [22]. On the other hand, low albumin not only weakens antioxidant and anti-inflammatory capacities but may also directly affect the supply of substrates required for wound healing [23]. Albumin is a key protein for maintaining plasma osmotic pressure and transporting nutrients, and its decline directly impacts the supply of amino acids and fatty acids necessary for tissue repair [24]. Additionally, low albumin is often accompanied by elevated levels of inflammatory cytokines such as IL-6 and TNF-α, which can further inhibit keratinocyte and endothelial cell function, delaying epithelialization and angiogenesis [25]. It is worth noting that the molecular pathological mechanisms of DFU involve various inflammatory factors and matrix metalloproteinases, which is mechanistically consistent with the inflammatory state reflected by BAR [26]. The role of IL-6 in wound healing is complex and context-dependent. IL-6 may signal through two major pathways: classical signaling and trans-signaling. In classical signaling, IL-6 binds to membrane-bound IL-6 receptor (mIL-6R) and subsequently activates gp130-mediated downstream pathways, including JAK/STAT and MAPK signaling. This pathway may exert reparative effects by supporting keratinocyte activation, epithelialization, and angiogenesis during the normal wound healing process. In contrast, IL-6 trans-signaling occurs when IL-6 binds to soluble IL-6 receptor (sIL-6R), allowing the IL-6/sIL-6R complex to activate gp130 on a wider range of cells. In chronic diabetic wounds, excessive or persistent IL-6 trans-signaling may promote leukocyte recruitment, endothelial activation, macrophage inflammatory polarization, and sustained production of inflammatory mediators, thereby delaying the transition from the inflammatory phase to the proliferative phase. Therefore, the effect of IL-6 on DFU should not be interpreted simply as harmful or beneficial, but rather depends on the balance between classical signaling and trans-signaling, the duration of cytokine exposure, and the inflammatory microenvironment [27].

This study also identified a non-linear relationship between BAR and DFU, with a *p*-value for non-linearity of 0.0127, suggesting that the effect of BAR on DFU risk is not simply linear but increases more significantly at higher levels. This finding differs slightly from the nearly linear relationship observed by Dai et al. in their study on diabetic retinopathy, which may reflect differences in the response patterns of different diabetic complications to BAR [19]. Future research could further explore the threshold effects of BAR across various complications and provide a basis for early intervention in high-risk patients.

It is noteworthy that, in addition to BAR, various novel biomarkers have recently been found to be closely associated with the healing process of DFU and diabetic complications. For instance, reduced serum levels of adipose triglyceride lipase are significantly correlated with renal impairment in diabetic kidney disease, reflecting the role of lipid metabolism disorders in diabetic complications [28]. Moreover, various inflammatory cytokines, growth factors, and matrix metalloproteinases play important roles in the pathological process of DFU [26]. These molecular markers may share overlapping or complementary mechanisms with BAR in reflecting inflammation, metabolism, and vascular dysfunction. Future research could explore the relationship between BAR and these molecular markers and even develop multi-indicator combined prediction models to further improve the accuracy of DFU risk assessment.

In terms of clinical translation, BAR offers several advantages: it is calculated from routine biochemical parameters, making it low-cost and easily accessible for healthcare institutions at all levels; its calculation is straightforward, allowing clinicians to quickly assess patient risk; moreover, BAR integrates multiple pathophysiological information, making it more comprehensive and representative than single indicators. Therefore, BAR is expected to become a powerful tool for risk stratification and early warning of DFU, particularly in resource-limited settings.

However, this study has several limitations. The diagnosis of DFU in this study was based on self-reported history of foot ulceration rather than clinically confirmed active DFU at the time of examination, which may introduce recall bias and misclassification. First, its cross-sectional design precludes the establishment of a causal relationship between BAR and DFU. Second, the diagnosis of DFU relied on self-reported questionnaires, which may introduce recall bias. Additionally, NHANES data did not include certain potential confounding factors such as ulcer severity, infection status, and local blood supply, which may affect the accuracy of the results. Future prospective cohort studies or interventional trials are needed to further validate the predictive value of BAR and explore the relationship between its dynamic changes and the progression of DFU. Another important limitation is that eGFR was not included as an additional adjustment variable in the present analysis. Because BAR is calculated using blood urea nitrogen and is therefore inherently influenced by renal function, the current results cannot fully determine whether the association between BAR and DFU is independent of renal impairment. Moreover, additional adjustment for eGFR may introduce potential overadjustment by removing part of the renal-function-related information captured by BAR. Therefore, our findings should be interpreted as demonstrating an association between BAR and DFU after adjustment for the covariates included in the current models, rather than proving complete independence from kidney dysfunction. BAR should not be interpreted as a replacement for conventional renal function markers such as serum creatinine or eGFR. Rather, BAR may serve as a composite biomarker integrating information related to blood urea nitrogen, albumin, nutritional status, and systemic inflammation. Further studies directly comparing BAR with standard renal function indicators are warranted. Future prospective studies incorporating eGFR, urinary albumin, and other renal function parameters are needed to validate whether BAR has incremental predictive value beyond conventional renal indicators. Additionally, several clinically important factors closely related to diabetic foot ulcer development, including peripheral arterial disease, diabetic neuropathy, other chronic diabetic complications, and detailed renal function parameters, were not included in the present analysis. The absence of these variables may have resulted in residual confounding and could have influenced the observed associations. Therefore, caution is warranted when interpreting the independent predictive value of BAR. Future prospective studies incorporating vascular, neurological, and renal assessments are needed to further validate our findings. Although triglycerides, total cholesterol, and HbA1c were included as covariates in the fully adjusted regression model, these glyco-metabolic parameters were not further evaluated in subgroup analyses. Because these variables are continuous indicators, categorizing them for subgroup analysis may lead to information loss and potential misclassification. Moreover, the limited number of DFU cases may increase the instability of estimates in multiple subgroup analyses. Therefore, whether glyco-metabolic status modifies the association between BAR and DFU requires further validation in larger prospective cohorts.

## 5. Conclusions

This study demonstrates that BAR is independently associated with a higher risk of DFU in a large-scale population. As a composite indicator integrating information on renal function, nutrition, and inflammation, BAR demonstrates good clinical applicability and potential for practical application. Future studies should further establish the critical value of BAR, validate its predictive performance in prospective cohorts, and explore its additional value when used in combination with other biomarkers such as inflammatory cytokines and lipid metabolism indicators. In this way, BAR is expected to become an important component in the early identification and intervention strategies for diabetic foot complications.

## Figures and Tables

**Table 1:** Basic characteristics of participants.

Characteristic	Overall, N = 1337 (100%) ^1^	Non-DFU, N = 1237 (92%) ^1^	DFU, N = 100 (8.2%) ^1^	*p* Value ^2^
Age, (years)	61.55 (11.60)	61.55 (11.69)	61.58 (10.59)	>0.9
Sex, n (%)				>0.9
Female	621 (47.52%)	582 (47.43%)	39 (48.58%)	
Male	716 (52.48%)	655 (52.57%)	61 (51.42%)	
Race				0.031
Mexican American	396 (7.46%)	365 (7.62%)	31 (5.71%)	
Other Hispanic	55 (5.26%)	49 (4.67%)	6 (11.80%)	
Non-Hispanic White	546 (67.32%)	504 (67.06%)	42 (70.25%)	
Non-Hispanic Black	291 (13.21%)	272 (13.48%)	19 (10.10%)	
Other Race	49 (6.76%)	47 (7.17%)	2 (2.15%)	
PIR	2.75 (1.54)	2.77 (1.55)	2.56 (1.51)	0.4
Education, n (%)				0.7
Below high school	629 (30.02%)	580 (30.22%)	49 (27.77%)	
High school or above	708 (69.98%)	657 (69.78%)	51 (72.23%)	
BMI, n (%)				<0.001
<30	693 (47.85%)	652 (49.23%)	41 (32.48%)	
≥30	644 (52.15%)	585 (50.77%)	59 (67.52%)	
Smoking status, n (%)				0.7
Current smoker	222 (18.95%)	203 (19.02%)	19 (18.15%)	
Former smoker	511 (37.53%)	474 (37.92%)	37 (33.25%)	
Never smoker	604 (43.52%)	560 (43.06%)	44 (48.60%)	
Drinking status, n (%)				0.5
No	539 (39.14%)	501 (39.48%)	38 (35.43%)	
Yes	798 (60.86%)	736 (60.52%)	62 (64.57%)	
Hypertension, n (%)				0.7
No	339 (27.39%)	316 (27.62%)	23 (24.94%)	
Yes	998 (72.61%)	921 (72.38%)	77 (75.06%)	
Triglyceride	206.08 (231.94)	207.35 (239.48)	191.94 (118.83)	>0.9
Total cholesterol	204.61 (47.51)	205.26 (47.75)	197.31 (44.24)	0.2
HbA1c	7.22 (1.65)	7.22 (1.66)	7.14 (1.50)	>0.9

^1^ Mean (SD); n (unweighted) (%): n represents the unweighted sample size, whereas percentages represent NHANES survey-weighted estimates. ^2^ Design-based Kruskal–Wallis test; Pearson’s X^2^: Rao & Scott adjustment. PIR: Income poverty ratio; BMI: Body Mass Index; Glycosylated hemoglobin: HbA1c. In Table 1, *p*-values for categorical variables with more than two levels represent the overall comparison of the distribution of the entire variable between the DFU and non-DFU groups using the design-based Rao–Scott adjusted chi-square test, rather than separate comparisons for each individual category level.

**Table 2:** Association between BAR and the risk of DFU.

	Model 1			Model 2			Model 3		
Characteristic	OR ^1^	95% CI ^1^	*p*-Value	OR ^1^	95% CI ^1^	*p*-Value	OR ^1^	95% CI ^1^	*p*-Value
BAR	1.17	1.09, 1.26	<0.001	1.21	1.08, 1.34	0.001	1.21	1.09, 1.35	<0.001
BAR quartile									
Q1	—	—		—	—		—	—	
Q2	0.29	0.09, 0.93	0.039	0.32	0.09, 1.06	0.062	0.30	0.09, 1.02	0.054
Q3	1.27	0.49, 3.26	0.614	1.44	0.52, 4.02	0.470	1.39	0.50, 3.87	0.518
Q4	2.50	1.21, 5.18	0.015	2.98	1.05, 8.50	0.041	3.11	1.09, 8.92	0.035

^1^ OR = Odds Ratio, CI = Confidence Interval. Model 1: No covariates were adjusted. Model 2: Age, sex, race, education, PIR, BMI, smoking, and drinking were adjusted. Model 3: Age, sex, race, education, PIR, BMI, smoking, drinking, hypertension, triglycerides, total cholesterol, HbA1c were adjusted. BAR was calculated as serum urea nitrogen divided by serum albumin. For categorical analysis, BAR was divided into quartiles according to its distribution among all included diabetic participants, and the lowest quartile was used as the reference group.

## Data Availability

Publicly available datasets were analyzed in this study. These data can be found here: http://www.cdc.gov/nchs/nhanes (accessed on 10 September 2025).
